# Unraveling Reading Achievement Through Educational Leadership, School Actions to Sustain Learning, Digital Self-Efficacy, and ICT-Related Factors: A Multilevel Mediation Analysis of PISA 2022 Türkiye Data

**DOI:** 10.3390/jintelligence14070137

**Published:** 2026-07-03

**Authors:** Nermin Er Aydemir, Ahmet Şahin

**Affiliations:** 1 Turkish Education Department, Faculty of Education, Alanya Alaaddin Keykubat University, 07425 Alanya, Türkiye; nermin.eraydemir@alanya.edu.tr; 2 Educational Management Department, Faculty of Education, Alanya Alaaddin Keykubat University, 07425 Alanya, Türkiye

**Keywords:** educational leadership, digital self-efficacy, ICT-related factors, multilevel structural equation modeling, PISA, reading achievement, sustainable development goals, sustainable learning, Türkiye

## Abstract

This study investigates the relationship between educational leadership, ICT-related factors, and students’ reading performance using the PISA 2022 dataset for Türkiye. Drawing data from 7250 students and 196 school principals, we employed multilevel structural equation modeling and Bayesian estimation. At the student level, between-school actions to sustain learning (SCHSUST) were significantly related to reading achievement. All student-level ICT variables—self-efficacy in digital competencies, practices regarding online information, and subject-related ICT use—significantly predicted reading achievement. Bayesian mediation analysis confirmed significant indirect relationships at student level, indicating that SCHSUST is associated with reading achievement primarily through ICT variables. Students’ economic, social and cultural status predicted reading achievement, indicating socioeconomic inequalities. At the school level, educational leadership (EDULEAD) has been found to be positively associated with preparedness for digital learning and school preparation for remote instruction. However, EDULEAD was negatively related to reading achievement. At the school level, all indirect relationships were insignificant. Furthermore, “economic, social and cultural status” and “academic school selectivity” emerged as the strongest predictors of reading achievement at their respective levels, indicating that the majority of reading inequality is due to substantial between-school inequality, in addition to the socioeconomic basis of digital inequality. Overall, the study highlights that meaningful ICT integration, rather than mere infrastructure provision, is associated with improved reading achievement.

## 1. Introduction

The integration of information and communication technology (ICT) in educational settings has gained significant attention, especially in revolutionizing teaching methodologies, providing hands-on training, and assessing students (Kong et al., 2022; Li et al., 2025; OECD, 2015). At the same time, digital technologies have gained a crucial role in developing learning processes by removing the barriers of time and geographical limitations to accessing learning content. They have become essential and prevalent instruments for many learning and teaching processes, such as language learning (Gałan, 2023), reading skills (Yu et al., 2023), and academic achievement (Acun Çelik et al., 2024).

Many existing studies examining the effects of digital technologies on students’ reading achievement address topics such as students’ ICT proficiency and the frequency of ICT use (Yu et al., 2023). However, the evidence is mixed. X. Hu et al. (2018) found using the Program for International Student Assessment (PISA) 2015 dataset that access opportunities to ICT at school were positively associated with students’ academic achievement, whereas access opportunities to ICT at home revealed a negative relationship. In the same study, ICT use for academic purposes was negatively associated with student performance, whereas ICT use of ICT for entertainment was positively associated. Xiao et al. (2019), on the other hand, found that ICT use in schools and ICT availability at home were inversely related to students’ reading scores. Kong et al. (2022) deemed that ICT is not directly related to academic achievement, but is related through cognitive-motivational factors such as perceived autonomy, competence, and interest. In another study, Luo et al. (2024) found a negative correlation between students’ ICT literacy and their performance in English digital reading. Similarly, students’ use of ICT-based social media at school was negatively correlated with digital reading achievement from PISA 2009 to PISA 2018 (J. Hu & Yu, 2021). Steffens (2014) found that German and Dutch students who frequently used ICT tended to have higher PISA exam scores, whereas Finnish students with low ICT use also tended to achieve higher PISA exam scores. Salmerón and Delgado (2019) emphasized that the use of digital technologies inside and outside of school may have harmful effects on academic achievement, reading skills, and learning (p. 465). Set against evidence that ICT may improve teaching and learning (e.g., Momčilović & Ninković, 2024; Şahin, 2019), the overall picture, although there has been considerable investment in educational ICT across both OECD and developing countries, remains limited and, at times, disputed (Trucano, 2005; UNESCO, 2009). These inconsistent findings make a closer, multilevel examination of how digital technologies relate to reading achievement all the more necessary.

“Quality education,” the fourth Sustainable Development Goal (SDG 4) of the United Nations, promotes inclusive, equitable quality education and lifelong learning opportunities for all individuals (United Nations, 2015) and emphasizes that all learners should achieve relevant and effective learning outcomes, including literacy. It also draws attention to improving the learning environments of schools, including access to ICT for educational purposes. Understanding how schools’ ICT infrastructure, educational leadership, and students’ digital self-efficacy shape reading achievement is therefore linked to SDG 4. The COVID-19 pandemic made this association more visible by exposing disparities in schools’ digital preparedness (OECD, 2023c). These are also linked to SDG 10, the goal of “reducing inequality within and among countries” (United Nations, 2015). In education, inequality manifests through differences among socioeconomic groups and types of schools regarding access to quality education, digital resources, and learning outcomes. Large-scale international assessments such as PISA consistently reveal that students’ socioeconomic backgrounds are among the strongest determinants of academic performance and highlight the persistence of educational inequality (OECD, 2023c). In Türkiye, the education system stands out because of significant differences between school types (e.g., Science High Schools versus Vocational High Schools, public schools versus private schools), among other factors. Thus, a range of factors, such as the academic selectivity of schools, socioeconomic status, and student–teacher ratios, can affect learning. By examining how ICT-related factors, educational leadership, and other school characteristics relate to reading achievement in Türkiye at both the student and school levels, this study provides empirical evidence relevant to SDG 4 and SDG 10.

School leaders’ efforts to improve teaching and learning have been linked to school effectiveness and student achievement (Robinson et al., 2008). School leadership occupies a central position among the factors associated with student achievement. That said, the literature indicates that school principals rarely shape outcomes directly. Their influence tends to work through the conditions they create for teaching and learning that is by improving the environment and conditions in which instruction takes place (Hallinger & Heck, 1998; Leithwood et al., 2020a; Spillane, 2018; Yalçın & Çoban, 2023). In particular, instructional leadership foregrounds the principal’s role in defining the school’s purpose, managing the curriculum, and establishing a learning-focused climate (Hallinger, 2011). In their meta-analysis, Robinson et al. (2008) found a weak but consistent relationship between leadership and student outcomes, with the strongest association emerging when leaders were directly involved in instruction and teacher development.

Although these findings suggest contradictory and unsettled results (e.g., Leithwood et al., 2020b; S. Zhang & Wu, 2025), context may play a decisive role (Spillane, 2018; Yalçın & Çoban, 2023). Hallinger’s (2011) model treats the context of leadership as one of its descriptive dimensions. Indeed, in centralized systems, school principals have limited room for maneuvering over curriculum, staffing, and budget, which may weaken the effect of leadership efforts on learning outcomes (Cansoy et al., 2025).

The COVID-19 Pandemic further increased the importance of school leaders during periods of remote instruction, and leaders have been expected to coordinate schools’ digital readiness, develop teachers’ technological skills, and ensure the continuity of learning (Harris & Jones, 2020). In centralized education systems such as Türkiye’s, the relationship between school leadership and learning outcomes in the post-pandemic context remains uncertain. For this reason, in this study, we consider educational leadership not as a direct lever on reading achievement, but rather as a school-level factor that may operate through institutional readiness for digital and remote learning.

International large-scale assessments, such as PISA, provide significant data for identifying multilevel factors related to students’ academic achievement. PISA exams assess students’ ability to tackle real-life challenges through their knowledge and skills. These exams evaluate reading achievement as well as mathematics and science skills by focusing on both individual and school-level factors such as the learning environment, school climate, socioeconomic status, and technology use (OECD, 2024a). However, the PISA 2022 reading achievement results present complex circumstances for Türkiye. Although Türkiye has shown significant progress in mathematics and science achievements in recent years in PISA exams, it has not demonstrated the same trend of success in reading performance (OECD, 2004, 2007, 2010, 2014, 2016, 2019, 2023c, 2024b). In the PISA 2022 exam, Türkiye’s reading achievement was below the OECD average (OECD, 2023c). A recent study employing merged Turkish TALIS–PISA 2018 data showed that teacher-level factors such as certification, sense of efficacy, and pedagogical innovation are significantly related to student achievement in reading, math, and science, demonstrating that teacher qualities are also associated with student achievement (Guo & Tian, 2026). This study, on the other hand, aims to examine, within the framework of a multilevel structural equation model, student- and school-level factors, such as school actions to sustain learning, school leadership, and ICT-related factors, which we believe may be related to students’ reading achievement, using Türkiye’s PISA 2022 student and school datasets.

## 2. Theoretical Framework

### 2.1. ICT Integration in Education

One aspect of ICT integration is especially consequential for learning: students’ level of digital competence. Digital competence is an important determinant of learning and academic success. Throughout, we use digital competence to refer to students’ perceived capability to use digital technologies effectively for learning purposes, including the ICTEFFIC index. The term ICT skills is used as a broader umbrella term for technology-related proficiencies, and digital literacy is retained only when reporting findings from prior studies that originally employed that terminology. Indeed, many studies have mentioned a link between students’ digital competence and their academic success (e.g., X. Hu et al., 2018; Yu et al., 2023). Therefore, the integration and effective use of ICT in teaching and learning processes is of great importance.

### 2.2. The Relationship Between Digital Technologies, Digital Learning Environments, and Reading Skills

Reading skills are regarded as a crucial area of competence for both academic achievement and lifelong learning. PISA reading achievement is a measure of students’ understanding, using, and reflecting skills on written texts (OECD, 2024b). Several variables, such as student characteristics and school-level factors, including school leadership, school environment, teaching processes, teacher competencies, and technological infrastructure, may play a significant role in enhancing reading achievement.

In recent years, the number of studies investigating the impact of ICTs on students’ academic achievement has increased (e.g., Kong et al., 2022; Luo et al., 2024; Yu et al., 2023). Furthermore, in digital learning environments, factors such as students’ technology usage skills, perceptions of digital self-efficacy, and behaviors regarding access to online information can influence a range of learning outcomes, including reading achievement. However, research indicates that student achievement has a multilevel structure; in addition to students’ individual characteristics, a variety of school-level factors, such as institutional characteristics, instructional leadership, resource allocation, and school policies, may also affect students’ technology usage styles and behaviors, learning processes, and ultimately, academic achievement (e.g., Xiao & Hew, 2022; D. Zhang et al., 2025). Therefore, student achievement is a pedagogical issue that must be addressed sensitively, considering both student- and school-level variables.

### 2.3. PISA 2022 ICT Framework and Digital Self-Efficacy

The PISA 2022 ICT Framework provides a useful theoretical framework for evaluating ICT integration into teaching and learning. This theoretical framework essentially consists of three components: (1) access to ICT (how students access ICT resources both inside and outside school), (2) ICT use (how students use ICT in educational processes and how teachers, schools, and education systems integrate ICT into pedagogical practices and learning environments), and (3) students’ ICT competencies (students’ digital literacy skill domains, as well as their attitudes and inclinations towards using ICT for learning and leisure). This framework enables an understanding of how system-level factors affect the ICT-related experiences of schools and students, how ICT availability and use interact with various teaching practices, and how these relationships correlate with students’ reading achievement (OECD, 2023a). This framework points out that ICT should be studied not only as a material that strengthens the physical infrastructure of schools but also as a pedagogical tool. Indeed, some research indicates that physical access to ICT alone is insufficient for explaining academic achievement; the ways in which ICT is used and perceived, along with students’ and teachers’ competence levels regarding ICT use, are also decisive factors (e.g., X. Hu et al., 2018; Skryabin et al., 2015). Therefore, we opted for the PISA 2022 ICT Framework as our study’s theoretical framework.

Another theoretical foundation of this study is the Self-Determination Theory (Deci & Ryan, 1985). This theory focuses on our experiences of choice as perceived internally by the individual. Our intrinsic growth tendencies and innate psychological needs form the foundations of our intrinsic motivation and personality integrity. According to self-determination theory, individuals’ intrinsic motivation increases when three basic psychological needs (autonomy, competence, and relatedness) are met. At the same time, these psychological needs contribute positively to learning and to educational outcomes associated with learning (Ryan & Deci, 2000). In the proposed model, among the student-level ICT variables, self-efficacy in digital competencies (ICTEFFIC) is associated with the competence dimension of self-determination theory, because this variable reflects students’ belief that they can complete learning tasks using digital tools. But we are more cautious about the variables: subject -related ICT use during lessons (ICTSUBJ) and students’ practices regarding online information (ICTINFO). Rather than establishing a direct link to self-determination theory, we consider ICTSUBJ and ICTINFO to be behaviors that can support need satisfaction. Subject-related ICT use can grant students a certain degree of freedom in how they approach learning tasks, which is consistent with autonomy in a broader sense. Students’ practices regarding online information, such as comparing resources and evaluating online content, involve interaction with a broader community of knowledge, and at times with peers and teachers, and in this respect loosely align with the dimension of relatedness. We acknowledge that these last two pairings are interpretive rather than definitive; therefore, we use self-determination theory not as a tool to rigorously test which need each variable fulfills, but as a general motivational lens to explain why digitally competent and engaged students may perform better.

### 2.4. Multilevel Nature of Student Achievement

Studies examining the relationships among ICT, school leadership, academic achievement, and reading achievement are common. However, most of these studies have been conducted using single-level analyses at the student level. School-level factors have generally been neglected in the literature. However, student achievement has a multilevel structure influenced by both individual and school-level variables. Multilevel modeling of student achievement, by considering the nested nature of the data, allows for the simultaneous and eclectic examination of variables at both the student and school levels. Thus, more valid and reliable results were obtained (Erer et al., 2025; Hox, 2010). However, multilevel studies examining student- and school-level variables related to student achievement remain limited (Erer et al., 2025). In particular, there are very few studies that investigate the joint effects of students’ digital skills and technology use behaviors, as well as school-level digital readiness and leadership factors—which are also the subject of this research—on students’ reading achievement. This highlights the need for studies that address the relationship between digital technologies, technology-supported learning processes, and students’ academic achievement from a multilevel perspective.

#### 2.4.1. Student-Level Variables and Reading Achievement

The actions taken by schools to sustain learning during the pandemic (SCHSUST) constitute the student-level independent variable in our model. The SCHSUST measures the precautions taken by schools to ensure the continuity of educational activities during school closures caused by the pandemic, as well as students’ perceptions of student well-being during this period (OECD, 2023b). This variable encompasses school practices that support learning, such as sending educational materials to students for independent study, tracking assignments, uploading materials to online education platforms, and conducting live virtual lessons (ETS & OECD, 2021b).

The first student-level mediating variable is self-efficacy in digital competence (ICTEFFIC), which shows how students feel about their ability to use digital tools to meet their learning goals (OECD, 2024a). Studies indicate that students with high digital self-efficacy engage with ICT more regularly in their educational pursuits (Huang et al., 2021) and perform better performance in addressing challenges within online settings (Abdolrezapour et al., 2023). Students’ practices regarding online information (ICTINFO) comprise critical behaviors, including trusting online information, comparing sources during online searches, questioning the accuracy of online content, and engaging in discussions (ETS & OECD, 2021a; OECD, 2024a). However, some studies have revealed a negative relationship between social interaction in ICT use and reading achievement (e.g., Xiao & Hew, 2022). The use of subject-related ICT in lessons (ICTSUBJ) measures how frequently digital resources related to lesson topics are used in class (OECD, 2023b). The use of information and communication technologies related to lesson content, both during and outside class, is positively associated with students’ reading achievement (Sanfo, 2023). Economic, social, and cultural status (ESCS) was included in the model as a student-level control variable. According to the PISA 2022 data, socio-economically advantaged students have higher reading scores than their disadvantaged peers (OECD, 2023c). At the same time, the fact that ESCS also affects ICT-related variables indicates the potential moderating effect of this variable.

#### 2.4.2. School-Level Variables and Reading Achievement

Researchers have long investigated the relationship between school leadership and student achievement. Hallinger and Heck (1998) addressed this relationship in three ways: direct effects, mediated effects, and reciprocal effects. Several studies largely support the mediated-effect model; that is, the school leader influences success not directly, but by shaping the school’s environment and conditions (Hallinger & Heck, 1998; Hallinger, 2011; Leithwood et al., 2020a; Robinson et al., 2008; Spillane, 2018; Yalçın & Çoban, 2023).

Leithwood et al. (2010, 2020b) expanded on this view with the “Four Paths” model, proposing that leadership influences learning through rational, emotional, organizational, and family paths. In our study, we incorporated the organizational pathway to enhance Hallinger and Heck’s (1998) mediated-effect model. The organizational pathway encompasses structural conditions such as the school’s structures, cultures, policies, standard operating procedures, teacher competencies, and technical support (Leithwood et al., 2010). Our study is grounded in the perspective of an indirect association between educational leadership and reading achievement. Therefore, we propose educational leadership not as a direct factor associated with reading achievement but as a school-level variable that operates through organizational conditions such as digital readiness (DIGPREP) and readiness for distance education (SCPREPAP). Therefore, in our model, the relationship between educational leadership and reading achievement was tested through indirect pathways mediated by these organizational readiness variables.

Educational leadership (EDULEAD) encompasses all educational processes, such as improving instructional processes in schools, supporting teachers, and enhancing the learning environment. Leadership behaviors aimed at integrating educational technologies into educational processes have emerged as important factors in improving learning environments, developing technological infrastructure, and preparing schools for innovation. In addition, school-level factors, such as the level of technical infrastructure for digital learning, teachers’ proficiency in using information and communication technologies, digital learning opportunities, and the accessibility of these opportunities, can also influence students’ digital learning skills and experiences. However, current research presents contrasting results regarding the effects of educational leadership on educational outcomes. The findings on the effect of leadership on school outcomes are inconsistent and contradictory. Papadakis et al. (2024) emphasized that educational leadership enhances student achievement by improving teacher performance. In parallel, Yalçın and Çoban (2023) emphasized that school leadership behaviors indirectly influence student achievement through variables such as collaboration with teachers, classroom instruction, and family involvement. At this point, we can argue that EDULEAD may influence student achievement through mediator variables such as digital preparedness and school readiness for remote teaching. Digital learning preparedness (DIGPREP) measures the level of digital technology integration in schools’ educational processes by considering the school’s digital infrastructure, teachers’ technology competencies, and technical support for ICT. During the COVID-19 pandemic, schools in many countries shifted to remote teaching. School preparedness for remote teaching (SCPREPAP) measures the preparations schools made for remote teaching during this period (OECD, 2023c). As control variables, the student–teacher ratio (STRATIO) and academic school selectivity index (SCHSEL) were included in the school-level model.

## 3. The Context of Türkiye

Türkiye has been among the countries participated in the PISA exams for many years, and its students’ varying reading achievement results across different periods have drawn attention. The country achieved its highest reading score in the PISA 2012 exam, and immediately afterward, it obtained its lowest score in PISA 2015 (OECD, 2014, 2016). Türkiye experienced a strong recovery in reading achievement in the PISA 2018 exam; however, it saw another decline in the PISA 2022 exam, which coincided with the post-COVID-19 pandemic period (OECD, 2019, 2023c). With these rises and falls in performance, Türkiye has performed below the OECD average reading scores (OECD, 2004, 2007, 2010, 2014, 2016, 2019, 2023c, 2024b). Similar to many countries worldwide, Türkiye closed schools during the COVID-19 pandemic, suspended in-person education, and transitioned to remote learning on 23 March 2020. Throughout the pandemic, classes were conducted periodically through in-person instruction; however, they were mostly conducted through remote learning (Çiftçi, 2022).

The pandemic sharpened ICT related issues and highlighted the need to strengthen schools’ technological infrastructure and digital readiness (Timotheou et al., 2022). The readiness of educational institutions for remote teaching, and the activities they carried out in this context were decisive factors in maintaining students’ continuity of learning and academic success. During the pandemic, from and after 23 March 2020, all schools in Türkiye, following centralized decisions, began education through television broadcasts; the digital education portal of the Ministry of National Education, called the Education Informatics Network, was enriched with online resources for course content, and on 14 April 2020, the EBA Live Lesson Classroom Feature was launched. Thus, online live lessons began to be held at all grade levels. Another major factor that played a crucial role in this process was teachers’ proficiency in using ICT, online learning platforms, and students’ access to technology. During the pandemic, in October 2020, school administrators started to receive training on “Remote Education and Technology Leadership.” Rather than being an intervention based on educational leadership theory, this training served as a centrally organized administrative support initiative aimed at managing the transition to remote learning during the pandemic. Additionally, in December 2020, a “Digital Literacy” guide was developed to improve teachers’ digital competencies. In some regions, the Ministry of National Education distributed computers and tablets to disadvantaged students to improve access to remote education (Çiftçi, 2022).

## 4. The Present Study and the Conceptual Model

**Figure 1:**
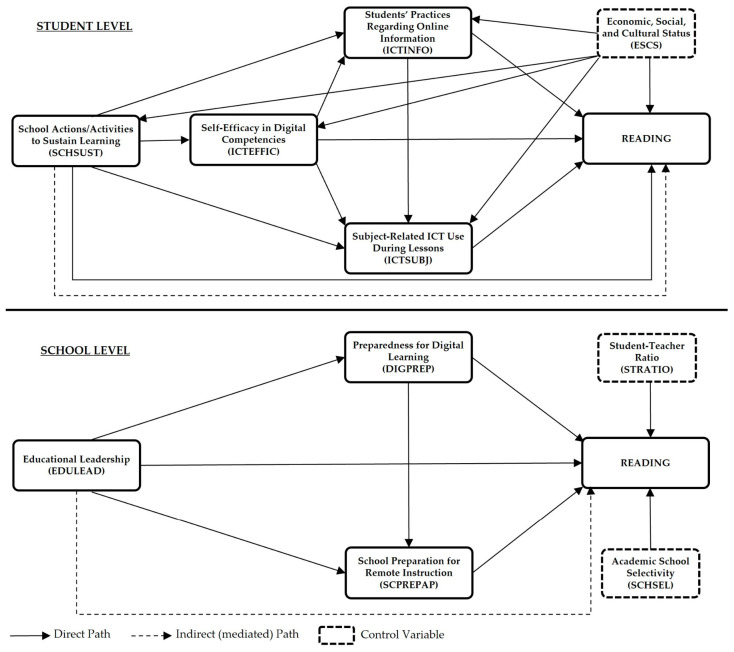
Conceptual multilevel model of direct and indirect associations of student- and school-level factors with reading achievement. Source(s): Authors’ own work.

This study aims to examine student- and school-level factors related to school leadership and ICT, which are believed to be associated with students’ reading achievement, using the PISA 2022 student and school datasets for Türkiye within the framework of a multilevel structural equation model. At the student level, we investigated the relationship between reading achievement and variables such as school activities aimed at continuing education, students’ perceptions of digital self-efficacy, their practices regarding online information, and technology use in lessons. At the school level, we focused on how factors such as educational leadership, preparedness for digital learning, and school readiness activities for remote teaching during the pandemic were related to reading achievement. Additionally, drawing from the literature, we used students’ family economic, social, and cultural status (ESCS) as a control variable in our student-level model. At the school level, we included the student–teacher ratio (STRATIO) and academic school selectivity (SCHSEL) as control variables in the model (Figure 1). That said, as there is no consistent theoretical or empirical basis to support a specific expectation regarding STRATIO, it was not linked to a hypothesis. In this context, this study primarily addresses the following research questions.

RQ1: At the student level, to what extent are school actions to sustain learning (SCHSUST), self-efficacy in digital competencies (ICTEFFIC), students’ practices regarding online information (ICTINFO), and subject-related ICT use during lessons (ICTSUBJ) directly and indirectly related to students’ reading achievement?

RQ2: At the school level, to what extent are educational leadership (EDULEAD), preparedness for digital learning (DIGPREP), and school preparation for remote instruction (SCPREPAP) directly and indirectly related to students’ reading achievement?

Drawing on our theoretical framework and along the axis of our research questions, we tested the following directional hypotheses as specified in our model:

**H1.**  *At the student level, school actions to sustain learning (SCHSUST) are positively associated with reading achievement, both directly and indirectly through self-efficacy in digital competencies (ICTEFFIC), students’ practices regarding online information (ICTINFO), and subject-specific ICT use during lessons (ICTSUBJ).*

**H2.**  *Each of the three student-level ICT variables (ICTEFFIC, ICTINFO, and ICTSUBJ) is independently and positively associated with reading achievement.*

**H3.**  *Economic, social, and cultural status (ESCS) is positively associated with reading achievement at the student level.*

**H4.**  *At the school level, educational leadership (EDULEAD) is positively associated with reading achievement, indirectly through preparedness for digital learning (DIGPREP) and school preparation for remote instruction (SCPREPAP).*

**H5.**  *Each of the school-level mediator variables (DIGPREP, and SCPREPAP) is independently and positively associated with reading achievement.*

**H6.**  *Academic school selectivity (SCHSEL) is positively associated with reading achievement at the school level.*

## 5. Materials and Methods

We employed a cross-sectional study design based on the PISA 2022 student and school datasets. In cross-sectional studies, data pertaining to the dependent, mediator, and independent variables are collected at a single point in time using the same questionnaire. The following section presents the study’s data, sample, measures, and data analysis strategy.

### 5.1. Data Source and Sample

Turkish datasets of student and school questionnaires from PISA 2022 were used in this study. A total of 7250 15-year-old students from Türkiye participated in PISA 2022. Of these students, 49.1% (*n* = 3561) were female, and 50.9% (*n* = 3689) were male. In addition, 196 school principals from Türkiye participated in the PISA 2022. Of these principals, 87.2% (*n* = 171) were working in public schools, while 12.3% (*n* = 24) were working in private schools. One principal did not report the school type. The average number of students enrolled in these schools was 696, and the average teacher–student ratio was 14.04. The average number of participants enrolled in each school was 36.98. At this point, Hox et al. (2010) suggested that the number of groups should be at least 50, and that the minimum number of participants per group for multilevel analysis should be 5. We argue that the clustered PISA 2022 dataset meets the requirements for a multilevel analysis.

### 5.2. Variables and Measures

This section introduces the variables and measures used in this study. We employed ten plausible variables and indices developed by the OECD as study measures. The OECD applied the Rasch model to scale dichotomous items and the partial credit model (PCM) to scale polytomous items and reported the resulting indices on a standardized metric (OECD mean = 0, SD = 1), where positive values indicated standing above the OECD average and negative values below it (OECD, 2023b). The Rasch model, a fundamental method in item response theory, is primarily used for binary items (such as yes/no or correct/incorrect) with a single difficulty parameter for each item. Its main advantages include ensuring measurement consistency and fairness across different groups, such as comparing students from various countries or backgrounds in tests such as PISA, detecting biases in questions through differential item functioning, and providing reliable results that are not sample-dependent, thus enhancing the trustworthiness and accuracy of scales used in education and psychology research (Wang & Wilson, 2005). The PCM used in PISA 2022 for constructing indices is a psychometric approach from item response theory that manages ordered, multi-category responses (such as Likert scales in questionnaires). It estimates an individual’s underlying traits (such as attitudes or behaviors) and extends the Rasch model by accommodating polytomous (multilevel) data with varying thresholds, making it more suitable for nuanced scales in PISA’s student, teacher, and school questionnaires (Wang & Wilson, 2005).

*Reading:* The dependent variable in this study is students’ reading proficiency, operationalized through the plausible values (PVs) from the PISA 2022 assessment. These PVs consist of ten imputed estimates (PV1READ to PV10READ) of each student’s latent reading ability, generated via item response theory scaling, primarily using the Rasch model for binary items and its extensions (e.g., partial credit model) for polytomous responses. Standardized with an OECD mean of 500 and a standard deviation of 100, the PVs account for measurement error and imputation uncertainty, enabling reliable multilevel analyses of reading achievement while preserving the full variance of the data (OECD, 2024a).

*School actions/activities to sustain learning (SCHSUST):* The independent variable of this study at the student level was the school’s actions/activities to sustain learning. We employed the SCHSUST index to measure our independent variable. This index measures students’ perceptions of the actions taken by schools during school closures (particularly due to COVID-19) to sustain learning and student well-being. This reflects the frequency and scope of the school’s interventions to ensure learning continuity, such as distributing learning materials, monitoring assignments, or conducting virtual classes. This index was constructed based on the ST348Q01JA-ST348Q08JA questions in the student questionnaire (ETS & OECD, 2021b).

*Self-efficacy in digital competencies (ICTEFFIC):* One of the mediator variables of this study at the student level was the students’ self-efficacy in digital competencies. We employed the ICTEFFIC index to measure this variable. It measures students’ perceptions of self-efficacy in performing various tasks using digital resources. This index reflects students’ confidence in skills related to information and communication technologies (ICT), encompassing digital competencies such as online information searching, data collection, programming, or collaboration. This index was constructed based on the IC183Q01JA-IC183Q16JA questions in the optional ICT familiarity questionnaire (ETS & OECD, 2021a).

*Students’ practices regarding online information (ICTINFO):* One of the mediator variables of this study at the student level was students’ practices regarding online information. We employed the ICTINFO index to measure this variable. It measures students’ practices related to online information. This index reflects students’ behaviors concerning digital information literacy, encompassing actions such as comparing sources during information searches, checking the accuracy of information, and discussing or flagging misinformation. This index was constructed based on the IC180Q01JA-IC180Q08JA questions in the optional ICT familiarity questionnaire (ETS & OECD, 2021a).

*Subject-related ICT use during lessons (ICTSUBJ):* One of the mediator variables of this study at the student level was students’ subject-related ICT use during lessons. We employed the ICTSUBJ index to measure this variable. It measures the frequency of students’ use of information and communication technologies (ICT) related to the subject matter during school lessons. This index reflects the extent to which students use digital devices for learning activities in specific subjects (e.g., mathematics, language, science, and other classes), encompassing tasks such as performing calculations, drawing graphs, running simulations, or conducting online research. This index is used to analyze the integration of digital tools in education and their relationship with student performance. This index was constructed based on the IC173Q01JA-IC173Q04JA questions in the optional ICT familiarity questionnaire (ETS & OECD, 2021a).

*Educational leadership (EDULEAD):* The independent variable of this study at the school level was educational leadership. We employed the EDULEAD index to measure this variable. It measures the frequency of school principals’ involvement in educational leadership activities. This index reflects the extent to which school management exhibited leadership behaviors, such as collaborating with teachers, providing information to parents, and resolving classroom discipline issues in the previous academic year (approximately, 12 months). This index was constructed based on SC201Q01JA-SC201Q11JA questions in the school questionnaire (ETS & OECD, 2021c).

*Preparedness for digital learning (DIGPREP):* One of the mediating variables in this study at the school level was preparedness for digital learning. We employed the DIGPREP index to measure this variable. It measures the capacity of schools to enhance learning and teaching using digital devices. This index reflects school administrators’ views on the school’s capacity regarding digital technology integration. It covers elements such as teachers’ skills, preparation time, professional resources, online platforms, incentives, and technical support. This index was constructed based on the SC155Q06HA-SC155Q11HA question in the school questionnaire (ETS & OECD, 2021c).

*School preparation for remote instruction in response to the pandemic (SCPREPAP):* One of the mediating variables of this study at the school level was school preparation for remote instruction in response to the pandemic. We employed the SCPREPAP index to measure this variable. It measures the extent of actions taken by schools to prepare for remote instruction during the pandemic (COVID-19). This index reflects the specific preparation activities for remote education in response to the pandemic, as reported by school administrators. It covers elements such as teacher and student training, material preparation, providing access, and transition planning. This index was constructed based on the SC223Q01JA-SC223Q10JA questions in the school questionnaire (ETS & OECD, 2021c).

*Control variables:* We used the index of economic, social, and cultural status (ESCS) at the student level as a control variable. The ESCS was derived from the standardization and weighted combination of data obtained from question groups in the PISA 2022 student questionnaire aimed at assessing parental education, parental occupation, and home possessions (ST251, ST254, ST255, ST005, ST006, ST007, ST008, ST014, and ST015) (ETS & OECD, 2021b).

We used the student–teacher ratio index (STRATIO) and the index of academic school selectivity (SCHSEL) at the school level as control variables. The STRATIO index in PISA 2022 measures the student–teacher ratio at the school level. This index reflects the number of students per teacher in schools and indicates the distribution of educational resources (in terms of teaching staff). This index was constructed based on the following questions: SC002Q01TA-SC002Q02TA and SC003Q01TA-SC003Q02TA. The STRATIO was calculated by dividing the total number of students by the full-time equivalent teacher count (ETS & OECD, 2021c).

The SCHSEL index in PISA 2022 measures the degree of selectivity in schools’ student admissions based on academic criteria. This index reflects how frequently schools consider academic performance and feeder school recommendations in their student admission policies on a categorized scale. This index was constructed based on the SC012 question groups in the school questionnaire (ETS & OECD, 2021c).

### 5.3. Analytical Strategy

This study employed the indices and 10 plausible values in reading produced by the OECD. Each plausible variable was used as the dependent variable in the study analyses, and the results of the 10 analyses were combined using Rubin’s pooling method. Rubin’s pooling method, also known as Rubin’s rules, is a statistical technique used to combine results from multiple versions of a dataset where missing data are filled in through a process called imputation. First, we took the average of the key findings or estimates from each imputed dataset to obtain an overall result. To measure the reliability of this average, we calculated the average amount of variation within each dataset, added the variation between the different datasets, and included a small adjustment to account for the uncertainty introduced by the imputation process itself. This approach ensured that our conclusions properly reflected both the data used and the guesses made for what was missing, leading to more reliable inferences. It is widely applied in analyses involving incomplete data to avoid underestimating the uncertainties (Jewsbury et al., 2024).

We conducted the data analysis using Mplus Version 8.3. The handling of incomplete data relied on Full Information Maximum Likelihood (FIML) combined with robust maximum likelihood estimation (MLR) to deliver estimates that withstand non-normal data patterns. This technique produces unbiased outcomes under the missing-at-random (MAR) condition (Muthén & Muthén, 2017).

To account for the complex sampling design of PISA 2022, the final student weight (W_FSTUWT) was included in the SEM analyses via Mplus’s WEIGHT command; thus, the parameter estimates reflected students’ unequal selection probabilities. Because W_FSTUWT already incorporates the school base weight (OECD, 2024a), no separate school weight was applied at the between-level. The nested data structure—the clustering of students within schools—was modeled through a two-level specification (CLUSTER = school ID; TYPE = TWOLEVEL), which partitions variance into within-school and between-school components and thereby accounts for the non-independence of observations. Standard BRR/Fay replicate weights are primarily designed for single-level variance estimation and are not directly combined with two-level structural models in Mplus (Muthén & Muthén, 2017). Therefore, we used a design-based MLR estimator that produces cluster-robust standard errors. Each of the 10 plausible values for reading was analyzed using this weighted two-level specification, and the results were combined according to Rubin’s rules. Bayesian analyses were conducted without sampling weights because the multilevel Bayesian framework in Mplus does not directly accommodate the PISA complex weighting procedure under a two-level specification (Muthén & Muthén, 2017). Thus, imputation uncertainty was incorporated throughout the study, and the sampling design was reflected in the weighted SEM estimates.

We employed a three-stage analysis. In the first stage, we conducted a preliminary analysis of the data. We calculated the arithmetic means, standard deviations, intraclass correlations (ICC), and Pearson correlation coefficients. We pooled the arithmetic means, standard deviations, and ICC values for the 10 plausible values in reading at this stage. These things considered, prior to modeling, all predictors were investigated for multicollinearity within a regression framework based on reading achievement. To do this, the tolerance and variance inflation factor (VIF) values were examined.

In the second stage, we conducted a multilevel structural equation modeling. In this regard, we reported the number of free parameters, chi-square value (χ^2^), degrees of freedom (df), *p*-value of the chi-square, scaling correction factor for MLR, Root Mean Square Error of Approximation (RMSEA), Comparative Fit Index (CFI), Tucker–Lewis Index (TLI), and Standardized Root Mean Square Residual (SRMR) (within-between) to assess model fit. Due to the sample size, the proposed model meets the acceptability criteria if its χ^2^/df falls at or under 5 (significant *p*-values presumed), RMSEA is capped at 0.07, SRMR is at or below 0.08, and CFI and TLI reach at least 0.92 (Hair et al., 2014). In this stage, we reported unstandardized and standardized estimates, errors and *p*-values. We pooled the model fit values, estimates, errors, and *p*-values at this stage.

Bayesian estimation was added to the frequentist SEM for the specific purpose of estimating indirect effects. Mediation is defined by the product of coefficients, and such terms rarely follow normal distribution. The Bayesian approach has a clear advantage. The credible intervals it produces are asymmetric, and they capture skewed effects more faithfully than the symmetric standard errors of frequentist estimators—a difference that matters in multilevel models in which the sampling distribution of an indirect effect is often visibly skewed (Miočević et al., 2018). Therefore, we base our inferences about the indirect pathways on the Bayesian estimates while using frequentist SEM for the direct effects and model-fit information.

In the third stage, we employed Bayesian estimation to examine indirect effects. Bayesian estimation offers advantages in handling complex hierarchical structures and providing full posterior distributions for parameter inferences. This approach involved specifying multilevel mediation models, where indirect effects were derived as the product of path coefficients (e.g., from predictor to mediator and mediator to outcome), with estimation conducted via Markov Chain Monte Carlo methods using non-informative priors for regression parameters and variance components to ensure flexibility and robustness, particularly in the presence of nested data, such as students within schools. The significance of indirect effects was assessed by examining whether the 95% credible intervals excluded zero, allowing probabilistic interpretations of mediation pathways (Miočević et al., 2018). However, given the cross-sectional design, these pathways reflect associations rather than causal relationships.

Estimation relied on Markov Chain Monte Carlo, using the Gibbs sampler together with Mplus’s default non-informative priors, so that the data—rather than any prior assumption—shaped the posterior. We checked for convergence in two ways: The Potential Scale Reduction factor (PSR) approached 1.0 for every parameter, and the trace plots told the same story: the chains were well mixed and showed no discernible trend.

We first reported the pooled versions of the number of free parameters, observed and replicated chi-square values, and posterior predictive *p*-value (PPP) to assess the model fit in Bayesian estimation. The observed and replicated chi-square values involving zero (0) and the PPP values between .40 and .60 are usually regarded as criteria for the goodness of fit (Gelman et al., 1996). We also reported pooled unstandardized estimates, posterior standard deviations, *p*-values, and lower and upper confidence intervals for the total, direct, and indirect paths in this stage.

## 6. Results

This section presents the study results. The results are presented in three subsections: preliminary analyses, structural equation modeling results, and Bayesian estimation results for the indirect effects.

### 6.1. Preliminary Analyses

This subsection presents the means, standard deviations, ICCs, Pearson’s correlation coefficients, tolerances, and VIF values. Table 1 presents the preliminary analysis results.

The reading scores in Table 1 show small-to-moderate associations with several student- and school-level variables. Among all predictors, students’ socioeconomic status (ESCS) demonstrated the strongest correlation with reading achievement (r = 0.34), indicating that a higher socioeconomic background was linked to higher reading achievement. Student-level ICT variables were also positively related to reading, although the effects were modest. Students who reported greater ICT use during lessons, stronger online information-handling practices, higher digital self-efficacy, and more school actions supporting learning showed slightly higher reading scores (rs = 0.18–0.23).

At the school level, the relationships with reading were generally weak. School selectivity showed the largest school-level association (r = 0.30), while digital preparedness and pandemic-related remote instruction preparation were positively but minimally correlated with reading achievement. Educational leadership displayed a small negative association with reading (r = −0.14).

The intraclass correlation coefficient (ICC) for reading was 0.51, indicating that roughly half of the variance in reading achievement lies between schools, reflecting substantial school-level differences. In contrast, student-level ICT variables showed low ICCs, suggesting that most of the variability in these measures occurred within schools. The ESCS index showed a higher ICC (0.34), indicating a notable clustering of socioeconomic status across the schools.

In addition, we calculated the tolerance and VIF values for multicollinearity concerns. The VIF values ranged from 1.025 to 1.155, while the tolerance values ranged from 0.866 to 0.976. These values are well below the common cutoff threshold (VIF < 10; tolerance > 0.10) and indicate that multicollinearity does not pose a problem in estimating the structural paths (Hair et al., 2014).

### 6.2. Structural Equation Modeling Results

We conducted 10 SEM analyses to examine the model fit and direct relationships between the study variables using each of the plausible values in reading as a dependent variable and then pooled the results following Rubin’s rules. The pooled model fit indices indicated a good fit to the data: number of free parameters = 38; chi-square value = 6.022; degrees of freedom = 4; *p*-value = .198; scaling correction factor for MLR = 0.810; RMSEA = 0.001; CFI = 0.999; TLI = 0.994; SRMR (within) = 0.000; SRMR (between) = 0.047. Table 2 presents the pooled SEM results for both student- and school-level analyses.

Table 2 presents the structural equation modeling results for student- and school-level predictors of reading achievement. At the student level, all four ICT-related variables showed significant positive direct associations with reading achievement. These results supported H2. Students’ practices regarding online information had the strongest standardized association (β = 0.080, *p* < .001), followed by self-efficacy in digital competencies (β = 0.062, *p* < .01), subject-related ICT use during lessons (β = 0.061, *p* < .001), and schools’ actions to sustain learning (β = 0.044, *p* < .01). These findings indicate that higher digital competence, greater engagement with online information, and more frequent pedagogical use of ICT each show a small but statistically reliable association with reading achievement.

The model also revealed a network of significant relationships among student-level predictors. SCHSUST positively predicted ICTEFFIC (β = 0.171), ICTINFO (β = 0.172), and ICTSUBJ (β = 0.133), suggesting that supportive school actions are associated with stronger student digital competence and greater ICT use in lessons. ICTEFFIC and ICTINFO were also positively associated (β = 0.299), and both contributed to ICTSUBJ. Socioeconomic status (ESCS) showed positive direct effects on reading (β = 0.065) and on all ICT-related variables (βs = 0.053–0.146), implying that students from higher socioeconomic backgrounds tended to report greater digital competence, more ICT-related practices, and, indirectly, higher reading achievement. Thus, H3 was supported.

At the school level, academic school selectivity (SCHSEL) demonstrated the strongest association with reading (β = 0.372, *p* < .001), indicating substantially higher reading scores in more selective schools, which supported H6. School preparation for remote instruction (SCPREPAP) was also positively associated with reading (β = 0.155, *p* < .05), whereas digital preparedness (DIGPREP) was not a significant predictor. Educational leadership (EDULEAD) exhibited a significant negative association with reading (β = −0.198, *p* < 0.01), suggesting increased leadership efforts in schools with relatively lower reading scores. Further, EDULEAD positively predicted both digital preparedness (β = 0.180) and pandemic-related school preparation (β = 0.170), and DIGPREP predicted SCPREPAP (β = 0.226), suggesting a coherent chain in which stronger leadership is linked to greater institutional readiness for digital and remote learning, even though these factors translate into only limited direct influence on student outcomes. Accordingly, H5 was partially supported, and the indirect pathways from educational leadership through DIGPREP and SCPREPAP were not significant (Table 3), indicating that H4 was not supported. Although H4 only concerned the indirect pathway, the analysis also revealed a significant negative direct association between leadership and reading (Table 2). Finally, the student–teacher ratio (STRATIO) had a small positive association with reading (β = 0.036, *p* < .01), indicating slightly higher reading achievement in schools with higher ratios.

### 6.3. Bayesian Estimation Results

We conducted a Bayesian estimation to examine the indirect effects. First, we checked the model fit. Similar to the SEM results, pooled model fit indices from Bayesian estimation indicated a good fit to the data: number of free parameters = 38; the 95% credible interval for the difference between observed and replicated chi-square values ranged from −24.104 to 25.713; posterior predictive *p*-value (PPP) = .440. Table 3 presents the Bayesian estimation results for indirect effects.

Bayesian mediation analysis revealed substantial and statistically credible indirect effects at the student level. School actions to sustain learning (SCHSUST) were positively and directly associated with students’ reading achievement (B = 2.647, 95% CI [0.689, 4.605]), indicating that Bayesian mediation analysis supported SEM analyses. SCHSUST was positively associated with students’ reading achievement through several ICT-related mediators. The strongest indirect pathways operated through students’ online information practices (ICTINFO; B = 0.818, 95% CI [0.475, 1.161]) and subject-related ICT use during lessons (ICTSUBJ; B = 0.482, 95% CI [0.237, 0.727]). Students’ self-efficacy in digital competencies (ICTEFFIC) also functioned as an important mediator (B = 0.634, 95% CI [0.270, 0.998]), both directly and through sequential pathways involving ICTINFO (B = 0.244, 95% CI [0.140, 0.348]) and ICTSUBJ (B = 0.039, 95% CI [0.014, 0.064]). A three-step chain—ICTEFFIC → ICTINFO → ICTSUBJ—showed a smaller yet credible effect (B = 0.025, 95% CI [0.011, 0.039]). All these student-level indirect paths had posterior *p*-values below .01, indicating high certainty in the Bayesian framework. The total indirect effect at the student level was large and credibly above zero (B = 2.253, 95% CI [1.751, 2.755]). Together with the significant direct path reported (β = 0.044) in Table 2, these significant indirect pathways through ICTEFFIC, ICTINFO, and ICTSUBJ supported H1.

In line with the SEM analyses, Bayesian mediation analysis revealed that educational leadership (EDULEAD) was negatively and directly associated with students’ reading achievement (B = −10.403, 95% CI [−17.614, −3.192]). In contrast, the indirect effects at the school level were more uncertain. Although the point estimates for the pathways from EDULEAD through digital preparedness (DIGPREP) and school preparedness for remote instruction (SCPREPAP) were positive, their credible intervals all included zero. The indirect effect via DIGPREP was small and highly uncertain (B = 0.223, 95% CI [−1.202, 1.648]; *p* = .380). The pathway through SCPREPAP was larger in magnitude but still not credibly different from zero (B = 1.249, 95% CI [−0.540, 3.038]). The sequential DIGPREP → SCPREPAP pathway also did not achieve statistical credibility. Consequently, the total school-level indirect effect (B = 1.872, 95% CI [−0.439, 4.183]) remained inconclusive.

Overall, the findings indicate that school actions aimed at sustaining learning operate primarily through students’ ICT-related competencies and use patterns, whereas indirect influences of educational leadership at the school level are less clearly supported by the data.

## 7. Discussion and Conclusions

Using PISA 2022 data for Türkiye, this study examined the potential relationship between students’ reading achievement, educational leadership, and ICT-related variables (SCHSUST, ICTINFO, ICTEFFIC, ICTSUBJ, DIGPREP, and SCPREPAP) at the student and school levels, employing multilevel structural equation modeling and Bayesian estimation methods. Considering the role of technology in education systems during the pandemic, our study extends previous research and is pertinent to understanding the role of educational leadership and ICT-related factors in promoting students’ reading achievement.

Regarding RQ1, the measures taken by schools in Türkiye to keep SCHSUST (Mean = −0.137) during the pandemic fell slightly below the OECD average. Furthermore, we found that all student-level ICT variables were statistically significant predictors of students’ reading achievement. ICTINFO had the strongest direct relationship with reading achievement. This was followed by ICTEFFIC, ICTSUBJ, and SCHSUST, respectively. This result confirmed the findings of Lee and Seo (2025) that students’ digital literacy and remote learning self-efficacy, such as online searching and help-seeking, enhanced their academic achievement (p. 139). In line with this, our findings indicate that higher digital competence, greater engagement with online information, and more frequent pedagogical use of ICT each show a small but statistically reliable association with students’ reading achievement. In other words, the study unravels the positive relationship between SCHSUST and students’ reading achievement, suggesting that the adoption of ICT-related factors is linked to students’ higher reading achievement. This pattern simultaneously underlines the need to incorporate students’ practices regarding online information and subject-related ICT use during lessons into learning practices to improve reading achievement. The results of this study are significantly aligned with recent research conducted by X. Hu et al. (2018), who highlighted the importance of ICT-related factors in student achievement. That study underscores that ICT skills, ICT access, and ICT use have a more positive effect on student academic performance. Furthermore, ESCS has a decisive relationship with reading achievement. Students from higher socioeconomic backgrounds tend to report greater digital competence, more ICT-related practices, and, indirectly, higher reading achievement. Likewise, Zheng et al. (2024) found a significantly positive relationship between ESCS and digital reading performance in most Asian regions at both the student and school levels. Kennedy and Strietholt (2023) found that the negative impact of school closures on students’ reading achievement was even more pronounced among socioeconomically disadvantaged students. Our findings indicate another problem related to the United Nations’ Sustainable Development Goals. In particular, SDG 4 and SDG 10 are directly related to our findings, particularly regarding ESCS, which was the variable most strongly associated with students’ reading achievement in Türkiye. This finding indicates inequality in education by highlighting that income inequality remains the greatest obstacle to students’ equal access to quality education in the country. It is worth emphasizing, however, that the magnitude of these ICT-related associations is modest. In standardized terms, they sit well below socioeconomic status and, at the school level, academic selectivity, which is by far the stronger correlate with reading achievement here. In other words, ICT operates at the margins rather than as a primary driver, and its practical weight should not be read as rivaling the structural inequalities that dominate the Turkish results. More broadly, the large analytic sample means that even very small coefficients attain statistical significance. Therefore, the significant ICT-related associations reported here should be read as statistically reliable but of modest practical and educational significance. In contrast ESCS and SCHSEL demonstrated substantially larger associations with reading achievement, suggesting that structural inequalities remain more influential than ICT-related factors.

Another important result that emerges from the student-level analysis is that this study extends the discourse by introducing ICTEFFIC, ICTINFO, and ICTSUBJ as significant indirect pathways, which introduces a nuanced understanding of how SCHSUST, beyond its direct influence, can be indirectly associated with improvements in reading achievement through its positive associations with ICTEFFIC, ICTINFO, and ICTSUBJ. The strongest indirect pathways were through students’ practices regarding online information (ICTINFO) and subject-related ICT use during lessons (ICTSUBJ). Students’ self-efficacy in digital competencies (ICTEFFIC) also functioned as an important indirect pathway, both directly and through sequential pathways involving ICTINFO and ICTSUBJ. This indicates that digital competence is a critical variable linked through indirect associations with how the actions taken by schools during the pandemic relate to students’ reading achievements. This finding concurs with Yu et al.’s (2023) argument that ICT-perceived competence and digital reading achievement are positively correlated and that ICT use significantly mediates this relationship. According to this study, students with higher-level ICT-perceived competence tended to use ICT for leisure at home more frequently, which led to better digital reading achievement. However, contrary to our findings, Motz et al. (2021) found that during the COVID-19 pandemic, as a result of remote learning, the number of assignments students received increased; naturally, they spent more time and exerted greater effort to complete these assignments; however, these students reported lower academic performance and felt less successful. They explain that this is because, under pressure to quickly transition teaching materials to remote instruction, assignments have turned into online tasks that do not constitute meaningful learning activities, which has negatively impacted student outcomes. Meanwhile, the mediating effects of ICTEFFIC, ICTINFO, and ICTSUBJ indicate that inequality increased in favor of students with access to digital tools, meaning that these students were more advantageous. We argue that the COVID-19 pandemic has widened this gap.

Regarding RQ2, the findings at the school level were more complex and striking. The usual expectation is that greater leadership efforts result in higher achievements. However, educational leadership exhibited a significant negative relationship with reading achievement, contrary to our hypothesis. Although this may seem contradictory at first glance, it may stem from the fact that school principals in Türkiye are primarily occupied with tasks such as paperwork, securing resources, and strengthening physical infrastructure rather than with instructional leadership. Similarly, a study conducted by Cansoy et al. (2025) found that school principals are unable to devote sufficient time to activities that enhance teaching and learning because they spend the majority of their time on administrative tasks (p. 1003). In short, the perception of successful leadership may indicate a heavy bureaucratic workload. Yet, principals in low-performing schools may increase educational interventions to boost success. Because the cross-sectional design cannot directly test the underlying mechanism, we read this coefficient as compatible with several non-exclusive explanations rather than as evidence of a substantive effect. First, the association may reflect reverse causality: principals in lower-performing schools may intensify leadership activity in response to weak results, so leadership is a reaction to low achievement rather than its cause. Cross-sectional data may have been collected during a period when the leader was exerting intensive efforts, and these efforts had not yet been reflected in student outcomes. That is to say, it may seem as though causality is operating in reverse, as the leader intervenes while the school’s reading achievement is already low. This is because cross-sectional studies cannot measure the influence of leadership over time (Hallinger, 2011). Moreover, school leaders do not cause failure; rather, they intervene because the school’s performance is low. Therefore, this situation indicates that, in cross-sectional PISA data, leadership is shaped according to the school’s context rather than causally. In other words, higher EDULEAD scores may partly reflect compensatory leadership efforts rather than the detrimental influence of leadership on student outcomes. Second, the EDULEAD index captures the reported frequency of leadership-related activities rather than the instructional quality or effectiveness of leadership practices. This index reflects the extent to which school principals exhibit leadership behaviors, such as collaborating with teachers, providing information to parents, and resolving classroom discipline issues (ETS & OECD, 2021c). Therefore, a higher score may not indicate more effective leadership. Third, school-level characteristics not modeled here, such as teacher quality, organizational climate, school resources, or students’ prior attainment, may also contribute to this negative association. Fourth, the centralized governance of the Turkish education system limits principals’ managerial authority. For example, school principals do not determine the curriculum or appoint teachers in the school. Their financial autonomy is also limited. Consequently, regardless of the strength of their educational leadership, they lack the autonomy to make radical decisions capable of transforming the system within schools. That is why leadership activities may be less directly connected to student outcomes than in more decentralized educational systems. This limited scope of the effect may partly explain why, in this context, stronger reported leadership does not correlate with higher reading achievements. Taken together, these considerations indicate that the negative relationship between EDULEAD and reading does not provide evidence that leadership reduces success, but rather a pattern that requires further investigation in future studies.

Furthermore, stronger educational leadership is linked to greater digital readiness and pandemic preparedness; however, neither of these readiness factors translates into a significant indirect relationship with students’ reading achievement. That is to say, stronger educational leadership is related to a greater institutional capacity, but this capacity does not show a corresponding link to students’ reading achievement. We do not consider this a shortcoming but rather an interpretable finding. Educational leadership may be related to reading achievement through mechanisms not included in our model, or readiness structures may operate at the institutional level within this centralized context rather than directly translating into academic benefits for students. The lack of support for H4 indicates that the school-level mediation model does not provide empirical support in this context.

SCPREPAP, which measures the extent of actions taken by schools to prepare for remote instruction due to the pandemic, was also positively associated with reading achievement, suggesting that teacher and student training, material preparation, providing access, and transition planning for remote instruction contribute to achievement-related outcomes. However, DIGPREP, which measures schools’ capacity to enhance learning and teaching with digital devices by evaluating factors such as teachers’ skills, preparation time, professional resources, online platforms, incentives, and technical support, was not a significant predictor of reading achievement. Therefore, H5 was partially supported: SCPREPAP was positively associated with reading, whereas DIGPREP was not. This pattern may indicate that having adequate digital infrastructure and resources in schools does not, by itself, contribute to improving learning outcomes. While DIGPREP addresses the capacity dimension of digital readiness, SCPREPAP reflects its implementation. Therefore, preparation processes based on concrete steps, such as teacher and student training, structured access planning, and instructional transitions, may have meaningfully contributed to students’ reading achievement. Furthermore, while countries with the shortest school closure periods during the COVID-19 pandemic experienced relatively less learning loss, those with the longest closure periods experienced greater learning loss (Jakubowski et al., 2025). Kennedy and Strietholt (2023) also expressed a similar view that there was a significant and substantial negative effect of school closures on student reading achievement. In this sense, we may argue that SCPREPAP plays a significant role in mitigating learning loss during remote learning processes in extraordinary situations, such as pandemics.

Regarding the controlling variables at the school level, the bivariate correlation between STRATIO and reading achievement was not statistically significant. This may be because correlation analyses treat the data as bivariate, that is, without controlling for other school-level variables or accounting for the nested data structure. However, because SEM simultaneously accounts for the nested data structure and controls for other school-level factors, it is a more robust type of analysis for revealing the true relationships at the school level. According to the results of the multilevel SEM, a small but significant relationship was observed in the multivariate model after controlling for other school-level factors, suggesting that the effect of STRATIO becomes evident only when the school context is considered. The small positive correlation between STRATIO and reading achievement contradicts the common expectation that smaller classes support learning. Therefore, it warrants a cautious reading. Even after SCHSEL is controlled, the residual variance in STRATIO may capture the school profile rather than the class size. Academically selective and high-performing schools (Science High Schools, exam-based Anatolian High Schools, Project Schools, etc.) in Türkiye are heavily oversubscribed and therefore comparatively crowded, while also enrolling academically successful students (MEB, 2025). A higher student–teacher ratio in these settings is thus a by-product of the demand for high-performing schools rather than a sign of under-resourcing (e.g., too few schools or teachers). Because school type is closely tied to selectivity and partly to staffing (e.g., the principals of Project Schools have partial authority over teacher recruitment), STRATIO may operate partly as a proxy for school type and selectivity rather than as a class size effect. Therefore, we treat the positive STRATIO coefficient as a pattern to be examined further and not as a substantive finding.

In addition, academic selectivity (SCHSEL) demonstrated the strongest relationship with reading achievement, indicating substantially higher reading scores in more selective schools. In fact, findings regarding school selection indicate that the type of school a student attends (Science High School, Exam-based Anatolian High School, Vocational High School, etc.) is the most significant factor determining reading achievement, rather than educational leadership, digital competence, digital readiness, or school support. Consequently, the school context—a school’s academic profile—may be a more decisive factor in students’ reading achievement than individual factors. This finding is also supported by the Intraclass Correlation Coefficient (ICC = 0.51). Accordingly, 51% of students’ reading achievement depends on the school they attend, reflecting substantial school-level inequality, which is also directly related to SDG 10. This is because the context of Türkiye’s educational landscape is unique and somewhat different. Students who take the PISA exams are generally high school students in Türkiye and are placed in high schools based on the results of the High School Placement Exam (LGS). Students with high academic achievement are primarily admitted to schools that select students based on exam scores, such as Science High Schools, exam-based Anatolian High Schools, and Social Sciences High Schools. Students who cannot gain admission to these schools—students with relatively lower academic achievement—are placed in high schools within their residential districts based on their home addresses or attend Vocational High Schools. Consequently, students’ academic achievement levels are a decisive factor in high school admissions. The reason why SCHSEL and ICC (0.51) may be related to reading achievement could stem from the fact that students in these schools already have high academic achievement.

## 8. Limitations and Practical Implications

The present study has limitations in several regards. First, this study has a cross-sectional design that utilizes data only from the PISA 2022 dataset for Türkiye. Since predictors, mediators, and the outcome variable were measured within the same PISA cycle, the results of the mediation analysis should be interpreted as model-based indirect relationships. Our results do not allow for causal inferences or generalization. Further research could be conducted based on datasets from other countries, including cross-country comparisons. In addition, the data were based on self-reports from students and school principals, which may have introduced potential measurement bias. To mitigate the measurement bias and limitations of the cross-sectional design, future research should conduct longitudinal designs to examine the temporal relationships between school actions to sustain learning, educational leadership, ICT-related factors, and students’ reading achievements, while also employing qualitative methods to thoroughly investigate context-specific mechanisms.

This study has several important implications. First, our findings offer valuable insights for developing measures and implementation strategies to address potential disruptions in education during future crises. In particular, schools should develop systematic and multifaceted strategies to ensure that learning continues during times of crisis, such as students’ and teachers’ digital competence and the use of ICT in the classroom, as well as preventing disparities stemming from socioeconomic status.

In addition to unqualified digital content and unplanned use of digital content, emphasis should be placed on practices that enable students to critically evaluate online information and use digital tools effectively for pedagogical purposes. School principals should not only focus on preparing digital infrastructure, but also on the pedagogical aspects of digital learning and remote instruction. That is, they should prioritize their instructional leadership roles. Finally, centralized education systems, such as Türkiye’s, should implement policy interventions to reduce digital inequality, particularly for socioeconomically disadvantaged students. Providing more qualitative support for technology-enhanced instruction, especially in disadvantaged schools, can reinforce the perceived positive effects of digital competence on students’ digital reading performance (Xiao & Hew, 2022; Yu et al., 2023).

Overall, this study reveals that the relationship between the measures schools took to sustain learning during the pandemic and reading achievement operates largely through indirect associations with students’ digital competence and ICT-related practices (ICTEFFIC, ICTINFO, and ICTSUBJ). This finding highlights the importance of access to remote education and digital pedagogy. Students’ practices regarding online information and the use of ICT in relation to course content are closely linked to the quality of digital pedagogy and represent a priority area that requires further attention. Furthermore, this study highlights both Quality Education (SDG 4) and Reduced Inequalities between and among Schools (SDG 10) by revealing that half of the variance in students’ reading achievement lies between schools. Socioeconomic status and academic school selectivity were the most significant factors. Our results indicate that educational inequalities cannot be resolved without meaningful ICT integration, rather than mere infrastructure provision.

Our findings also yield a practical implication for the training of school principals. In this study, we found that educational leadership was negatively related to reading achievement, but this likely reflects a role dominated by administrative workload and resource management demands rather than instruction. Read this way, the result points less to leadership being unhelpful than to the wrong kind of leadership being measured. A reasonable response, then, is to shift principals’ preparation towards instructional leadership. However, our results also caution against expecting too much from training alone: in a centralized system, structural constraints on principals’ autonomy may hinder even well-developed instructional capacity. Therefore, strengthening instructional leadership should to be considered alongside policy reforms that grant principals greater decision-making authority.

## Figures and Tables

**Table 1:** Means, standard deviations, ICCs, and Pearson’s correlation coefficients.

Variables	M	SD	ICC	1	2	3	4	5	6	7	8	9	10
**Dependent V.**													
1. Reading	451.163 ^†^	87.225 ^†^	0.508 ^†^	-									
**Student-Level**													
2. SCHSUST	−0.137	1.017	0.066	0.182 ***	-								
3. ICTEFFIC	0.006	1.118	0.047	0.194 ***	0.175 ***	-							
4. ICTINFO	0.151	1.145	0.043	0.210 ***	0.224 ***	0.335 ***	-						
5. ICTSUBJ	0.093	1.062	0.123	0.234 ***	0.186 ***	0.153 ***	0.200 ***	-					
6. ESCS	1.228	1.165	0.344	0.337 ***	0.131 ***	0.168 ***	0.128 ***	0.159 ***	-				
**School-Level**													
7. EDULEAD	0.566	1.098	-	−0.143 ***	−0.048 ***	−0.018	−0.019	−0.034 **	−0.050 ***	-			
8. DIGPREP	0.361	0.962	-	0.054 ***	0.018	0.044 ***	0.018	0.048 ***	0.095 ***	0.189 ***	-		
9. SCPREPAP	0.212	0.921	-	0.103 ***	0.002	0.027 *	0.036 **	0.020	0.084 ***	0.210 ***	0.253 ***	-	
10. STRATIO	14.042	4.740	-	0.019	0.037 **	0.078 ***	0.004	−0.006	−0.039 **	0.023 *	0.131 ***	−0.013	-
11. SCHSEL	2.260	0.846	-	0.297 ***	0.058 ***	0.001	0.051 **	0.069 ***	0.178 ***	−0.086 ***	0.043 ***	0.041 **	−0.023 *

Notes: ^†^ = Pooled values; * = *p* < .05; ** = *p* < .01; *** = *p* < .001; SCHSUST: School actions/activities to sustain learning; ICTEFFIC: Self-efficacy in digital competencies; ICTINFO: Students’ practices regarding online information; ICTSUBJ: Subject-related ICT use during lessons; ESCS: index of economic, social, and cultural status; EDULEAD: Educational leadership; DIGPREP: Preparedness for digital learning; SCPREPAP: School preparation for remote instruction in response to the pandemic; STRATIO: student–teacher ratio index; SCHSEL: index of academic school selectivity.

**Table 2:** SEM results regarding student- and school-level analyses.

Paths	B	S.E.	β (Standardized)	S.E. (Standardized)	*p*
**Student-Level**					
SCHSUST → Reading	2.647	0.968	0.044	0.016	.007 **
ICTEFFIC → Reading	3.394	0.980	0.062	0.018	.001 **
ICTINFO → Reading	4.274	0.843	0.080	0.015	.000 ***
ICTSUBJ → Reading	3.513	1.000	0.061	0.017	.000 ***
SCHSUST → ICTSUBJ	0.139	0.018	0.133	0.017	.000 ***
ICTEFFIC → ICTSUBJ	0.062	0.013	0.065	0.014	.000 ***
ICTINFO → ICTSUBJ	0.127	0.016	0.080	0.015	.000 ***
SCHSUST → ICTEFFIC	0.189	0.016	0.171	0.015	.000 ***
SCHSUST → ICTINFO	0.193	0.018	0.172	0.016	.000 ***
ICTEFFIC → ICTINFO	0.306	0.017	0.299	0.016	.000 ***
ESCS → Reading	3.450	0.931	0.065	0.018	.000 ***
ESCS → SCHSUST	0.122	0.013	0.140	0.015	.000 ***
ESCS → ICTEFFIC	0.141	0.012	0.146	0.013	.000 ***
ESCS → ICTINFO	0.052	0.012	0.053	0.012	.000 ***
ESCS → ICTSUBJ	0.102	0.016	0.112	0.017	.000 ***
**School-Level**					
EDULEAD → Reading	−10.411	3.741	−0.198	0.071	.005 **
DIGPREP → Reading	1.754	4.719	0.030	0.070	.672
SCPREPAP → Reading	9.515	4.220	0.155	0.071	.028 *
EDULEAD → DIGPREP	0.160	0.065	0.180	0.073	.014 *
EDULEAD → SCPREPAP	0.145	0.049	0.170	0.055	.002 **
DIGPREP → SCPREPAP	0.218	0.078	0.226	0.073	.002 **
STRATIO → Reading	0.029	0.010	0.036	0.013	.005 **
SCHSEL → Reading	25.086	4.385	0.372	0.060	.000 ***

Notes: * = *p* < .05; ** = *p* < .01; *** = *p* < .001; SCHSUST: School actions/activities to sustain learning; ICTEFFIC: Self-efficacy in digital competencies; ICTINFO: Students’ practices regarding online information; ICTSUBJ: Subject-related ICT use during lessons; ESCS: Index of economic, social, and cultural status; EDULEAD: Educational leadership; DIGPREP: Preparedness for digital learning; SCPREPAP: School preparation for remote instruction in response to the pandemic; STRATIO: Student-teacher ratio; SCHSEL: Academic school selectivity.

**Table 3:** Bayesian estimation results regarding indirect effects.

Paths	B	S.E.	Lower CI	Upper CI	*p*
**Student-Level**					
SCHSUST → Reading ^†^	2.647	0.991	0.689	4.605	.004 **
SCHSUST → ICTSUBJ →Reading	0.482	0.125	0.237	0.727	.000 ***
SCHSUST → ICTINFO → Reading	0.818	0.175	0.475	1.161	.000 ***
SCHSUST → ICTEFFIC → Reading	0.634	0.186	0.270	0.998	.000 ***
SCHSUST → ICTEFFIC → ICTINFO → Reading	0.244	0.053	0.140	0.348	.000 ***
SCHSUST → ICTEFFIC → ICTSUBJ → Reading	0.039	0.013	0.014	0.064	.001 **
SCHSUST → ICTEFFIC → ICTINFO → ICTSUBJ → Reading	0.025	0.007	0.011	0.039	.000 ***
Total Indirect Effects	2.253	0.256	1.751	2.755	.000 ***
**School-Level**					
EDULEAD → Reading ^†^	−10.403	3.679	−17.614	−3.192	.002 **
EDULEAD → DIGPREP → Reading	0.223	0.727	−1.202	1.648	.380
EDULEAD → SCPREPAP → Reading	1.249	0.913	−0.540	3.038	.086
EDULEAD → DIGPREP → SCPREPAP → Reading	0.283	0.249	−0.205	0.771	.128
Total Indirect Effects	1.872	1.179	−0.439	4.183	.056

Notes: ^†^ = Direct effect; ** = *p* < .01; *** = *p* < .001; SCHSUST: School actions/activities to sustain learning; ICTEFFIC: Self-efficacy in digital competencies; ICTINFO: Students’ practices regarding online information; ICTSUBJ: Subject-related ICT use during lessons; EDULEAD: Educational leadership; DIGPREP: Preparedness for digital learning; SCPREPAP: School preparation for remote instruction in response to the pandemic.

## Data Availability

Restrictions apply to the availability of these data. Data were obtained from PISA 2022 Database and are available from the website: https://www.oecd.org/en/data/datasets/pisa-2022-database.html (accessed on 22 January 2026).
